# Some attributes of pasture-based systems as they relate to dairy cow welfare

**DOI:** 10.3168/jdsc.2025-0947

**Published:** 2026-05-22

**Authors:** M.W. Fisher, J.R. Roche, K.E. Schütz, M. Verdon, L.M. Hemsworth

**Affiliations:** 1 Kotare Bioethics, Wellington, 6037, New Zealand; 2 School of Biological Sciences, University of Auckland, Auckland, 1142, New Zealand; 3 New Zealand Institute for Bioeconomy Science (AgResearch Group), Hamilton, 3240, New Zealand; 4 Tasmanian Institute of Agriculture, University of Tasmania, Tasmania, 7320, Australia; 5 Animal Welfare Science Centre, University of Melbourne, Victoria, 3010, Australia

## Abstract

Like all dairy cattle, pasture-based cows face challenges to their welfare, but there are also unique challenges because of the physical environment, which involves exposure to the vagaries of climate and an associated variability in feed supply and composition. Cows balance energy intake with energy expenditure, producing more milk when more pasture is available or when supplementary feed is provided, and less milk when feed is limited. The physiological balancing of milk production with nutrient supply may mean that dairy cows experience short-term fluctuations of hunger and satiety. Though generally having greater freedom for the expression of natural behaviors and often experiencing better health, exposure to the weather brings its challenges. Welfare may be compromised by excessive heat in summer and cold and wet conditions in winter. The provision of shade to protect cows from solar radiation and shelter to protect them from wind and rain, as well as access to clean, dry, and soft lying surfaces, can sometimes be a challenge, especially in large herds. Although the desire to provide livestock with shelter is acknowledged by producers, it is subject to resource availability and affordability and adequate information on relevant technologies and initiatives. Other technologies specific to pasture-based systems include virtual fencing and herding, where grazing and guiding of cows can be controlled by audio or vibration cues which, if ignored, are followed by an aversive electric shock. Safety mechanisms are required to ensure the animal can predict and control its circumstances and respond accordingly. Although they may improve labor efficiency or potentially provide more time for meaningful tasks and interactions with animals, such technologies may also alter how animals and humans interact, challenging traditional ideals of the human–animal relationship. In conclusion, alongside more traditional perspectives of what animals are experiencing, animal welfare critiques also need to acknowledge the dynamic and complex factors determined by human needs and expectations of animals and their husbandry.

## Highlights

• Pasture-based dairying is aligned with the seasons animals experience and adapt to.
• Shelter, technologies, and husbandry provide opportunities to enhance cow welfare.
• Animal welfare also depends on the interactions that individuals and societies justify.

## Graphical Abstract

Pasture-based dairy cows face unique challenges, being exposed to the vagaries of climate and an associated variability in feed supply and composition. Cows balance energy intake with energy expenditure, producing more milk when more feed is available and less when feed is limited. Solar radiation in summer may require the provision of shade. Shelter from wind and rain and access to comfortable lying surfaces may be subject to resource availability and affordability. Virtual fencing and herding controlling grazing and guiding herding may improve labor efficiency, and potentially animal health and welfare, though require that the animal be able to predict and control its circumstances and respond accordingly. Such technologies may also alter how animals and humans interact, challenging traditional ideals of the human-animal relationship, which is itself partly determined by the actions and attitudes of stockpersons.

**Figure:**
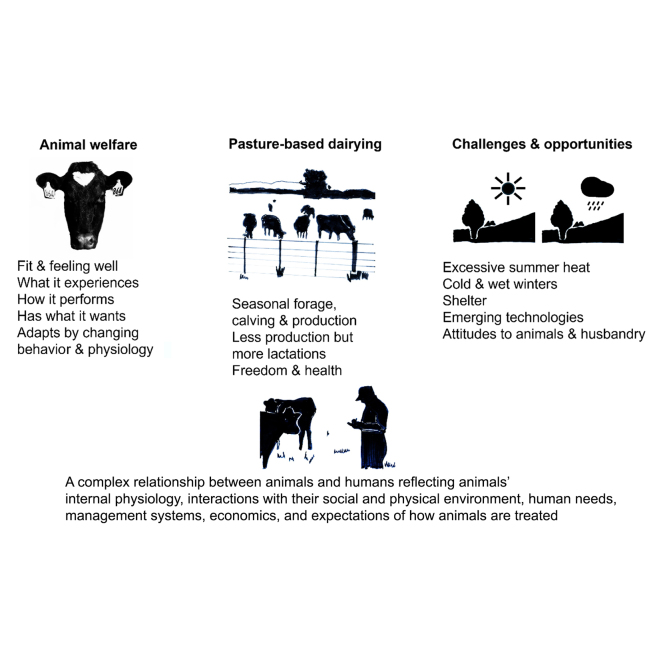

The relationship between animals and humans is long, complex, diverse, and enduring (Fisher, 2018, 2020), with co-dependence evident in the ancient contract, “we take care of the animals, and they take care of us” (Kilgour, 1985; Rollin, 2008). Part of what makes this relationship so valuable is that animals adapt, at least to some degree, to a range of human-centered environments and practices. For example, dairy cattle may be kept in intensive housing or in pastoral environments, be fed total mixed rations or primarily graze pasture, be kept in small or large herds, or be subject to husbandry reliant on traditional knowledge or complemented with automated and precision farming methods. However, lack of resources (including available husbandry skills), diseases, and other conditions can also compromise cows exposed to such environments and practices (von Keyserlingk et al., 2009; EFSA Panel on Animal Health and Animal Welfare et al., 2023; Verdon and Beggs, 2024). Although the challenges discussed in this review are broadly relevant across pasture-based grazing systems globally, their expression and severity may vary by geographic context, including climate, soil type, age, breed or genotype, nutritional management, and system-level practices, such as access to shade or shelter.

Contemporary concern for the care of animals is commonly expressed in terms of animal welfare, though the important reality that animals provide for humans is perhaps often overlooked. Reflecting the diversity of the relationship, there are a range of understandings of animal welfare (Fraser, 2003; Fisher, 2009; Rault et al., 2025), as there are expectations for the care and use of animals, for instance, what the animal experiences, whether it is fit, healthy, and feeling good, has what it wants, and performs well. Increasingly, such understandings are inferred from objective measures such as body condition and milk production, and behavior and physiological indices, as well as concepts such as whether the animal's circumstances are in keeping with its nature, what it has evolved to deal with, and even if it is treated with dignity. In other words, animal welfare depends on what the animal experiences.

Welfare is commonly expressed in terms of the “5 freedoms” (Webster, 2005) and subsequently as the physical and mental domains (Mellor and Beausoleil, 2015) of welfare (for example, nutrition, companionship, and shelter). Animal welfare science (e.g., Mellor et al., 2009) and stockmanship (e.g., Gatward, 2001) describe animal welfare through an animal lens. However, an animal's welfare also depends on the compromises society accepts as necessary and reasonable in satisfying human needs (for example, the provision of food, labor, and commerce), as well as the broader human social preferences and prejudices (for example, “we love some, hate some, and eat some”; Herzog, 2010).

Although it is primarily a reflection of the actions of farmers and their staff, including their preferences, principles, and circumstances (McInerney, 2004), farm animal welfare is increasingly influenced and determined by the expectations of the wider community. Complementing the animal lens, this additional social or cultural lens (Fraser, 2008; Fisher, 2018) reflects the interdependence of humans and domesticated farm animals. While acknowledging that there are many challenges to animal welfare within pasture-based production systems, the aim of this review is to reflect on some of the challenges associated with pasture living: nutrition and hunger, the provision of shade and shelter, managing the range and movement of animals at pasture, and the attitudes and behaviors of stockpeople.

Pasture-based systems, particularly those where the cows' diet during lactation is primarily obtained from grazed pasture and upon which cows spend most of their time (Horan et al., 2026) have some features in common with other dairy production systems and some distinctive features. Most obviously, the animals derive all or most of their nutritional needs from pasture and must undertake work to do so. In most pasture-based systems, energy demands are aligned with pasture supply by concentrating calving in late winter/early spring in order to maximize the utilization of seasonal pasture production and the provision of the most digestible feed during peak lactation (Spaans et al., 2019).

Animals deriving all or most of their nutritional needs from pasture typically produce less milk per day and per lactation than those in housed systems (Kolver and Muller, 1998), though they also have a greater average number of lactations. Because animal production is directly linked to DMI, low production could be viewed as an indicator that pasture-based animals are hungry (i.e., have a lower DMI when they could eat more). It could, therefore, be argued that pasture-based cows have less than optimal welfare compared with housed cows or grazing cows consuming nonpasture supplementary feeds. Such a simplistic conclusion misrepresents the complexity of hunger and satiety, however, as well as the neuroendocrine physiology underpinning signals to eat and cease eating.

Notwithstanding the obvious compromises to animal welfare associated with prolonged periods of underfeeding or severe malnourishment (Phillips, 2016), it is accepted that short-term periods of preprandial hunger along with periods of postprandial satiety are parts of the natural physiology all animals experience. Some of this is learned behavior, but mostly it is underpinned by hormonal and neural signaling (Roche et al., 2008). Hunger and satiety are primarily governed by neural circuits in the brain, with the hypothalamus serving as the central regulator of nutrient and water balance (Baile and McLaughlin, 1987; Roche et al., 2008).

Physiological mechanisms controlling DMI are intertwined with mechanisms regulating energy expenditure, ensuring that changes in energy or nutrient output (e.g., milk production) are matched by changes in energy or nutrient input (i.e., DMI). The push-pull system of greater demand resulting in increased DMI is demonstrated in early lactation, wherein DMI lags behind energy and nutrient requirements; the deficit is initially filled by mobilization of body reserves and bone resorption (Baile and Della-Fera, 1981; Roche et al., 2009). This early lactation deficit creates a physiological feedback loop to instruct the cow to eat more. The resultant greater DMI, in turn, enables greater milk production, and the increase in output drives the cow to increase DMI further, as the cow's brain perceives a higher requirement. The underlying mechanisms allow dairy cows to adapt their needs to their resources.

Pasture-based cows have lower DMI than housed cows fed a total mixed ration (Kolver and Muller, 1998). At least some of the reason for this is the time associated with grazing, as pasture-fed cows indoors have a greater DMI than grazing cows (Boudon et al., 2009). Similarly, grazing cows offered supplementary feeds have a higher DMI than unsupplemented grazing cows, although the consumption of the nonpasture feeds results in a reduction in pasture DMI (Bargo et al., 2003; Roche, 2017).

The physiology underpinning DMI is reported by Roche et al. (2026) and will not be discussed in detail here, but in both situations mentioned, when DMI is greater, milk production is also greater, with the increase being commensurate with the increase in intake of metabolizable energy (Kolver and Muller, 1998), assuming no other limiting variable (Roche, 2017). Because milk production increases with DMI, there is no difference in the nutrient/energy balance, and thus unsupplemented pasture-based cows should be no more or less hungry than supplemented pasture-based cows (Roche et al., 2026).

Consistent with the premise that production adjusts to accommodate DMI, Roche et al. (2007) investigated a range of farm system changes that affect DMI and milk production. They reported a positive relationship between milk production and DMI, but more importantly, the system-level effects on DMI and production had almost no effect on body condition: increased nutrient supply resulted in an equivalent increase in milk production (nutrient demand). These results suggest that the “push-pull” system of nutrient demand driving DMI (i.e., supply) and DMI driving greater production (i.e., demand) finds an equilibrium. It can therefore be inferred that short-term fluctuations in feelings of hunger and satiety associated with differences in DMI re-equilibrate, with animals with higher DMI producing more milk and, as a result, experiencing no less hunger than animals with a lower DMI, within normal ranges.

In short, the relationship between DMI, productivity, and the cow's experience of hunger and satiety is determined by neuroendocrine mechanisms that operate as a balancing feedback system: the drive to eat adjusts to match energy output, and vice-versa (Baile and Della-Fera, 1981). Modern dairy cows provided with additional feed will partition most of those nutrients and energy into producing extra milk rather than into excess body fat (Roche et al., 2006, 2009; Macdonald et al., 2008). In effect, the central nervous system balances DMI against production and, therefore, feelings of hunger and satiety. Although these insights pertain to the slightly lower production of cows on pasture, and not severely malnourished and low-producing animals, there are nevertheless aspects that require further understanding. These include (1) what feelings the cow experiences when adjusting DMI and expenditure and in the adjustments between new states; the influence of short-term increases in pasture DMI, through offering greater pasture allowances, compared with the consequent reduction in pasture availability and quality in later meals (i.e., improving nutrient intake at one point only to reduce subsequent nutrient availability; Stakelum and Dillon, 1990; Macdonald and Roche, 2024); (2) the impacts of different activities, especially walking between pasture and milking, which can be significant distances in pastoral systems; (3) the energy availability and requirements associated with adverse climatic conditions; and (4) the extent to which compromises can or cannot be justified or, if significant, mitigated. Thus, there are gaps in our knowledge of the affective experience of hunger during short-term fluctuations in feeding motivation in the absence of feed. Along with more nuanced perspectives of hunger, body condition, a measure of an animal's energy and protein reserves, is another nutrition-based variable arguably deserving of more sophisticated analysis and understanding relevant to animal welfare (Roche et al., 2009; Matthews et al., 2012; Fisher et al., 2020).

Pasture-based dairy cows are exposed to, and required to cope with, the vagaries of daily and seasonal fluctuations in climatic variables, as well as occasional extremes. There are also times when animal health, productivity, and welfare can be compromised (Hendriks et al., 2025). Shade is a valuable resource, and animals aggressively compete for it when access is limited (Schütz et al., 2010). However, not all shade is equal, with cows preferring and using shade more when it reduces greater levels of solar radiation (Schütz et al., 2009; Tucker et al., 2008). When cows are given more shade, their thermoregulatory abilities are greater (Schütz et al., 2010). For example, although animals could physically fit (stand) under 2 m^2^ of shade per cow, use of shaded areas increases with increasing shade availability, up to 15.6m^2^/cow, with fewer cows observed panting (Schütz et al., 2014). Lying under shade becomes more common when more shade is provided (Tresoldi et al., 2017), suggesting that shade recommendations should take into consideration how the resource is used (i.e., meaningful to the animals). Although shade can be provided by trees or a range of fixed or portable structures, with large herd sizes, it can be a substantial challenge to provide enough shade for all cows, especially in systems such as rotational grazing, in which cows are moved to new areas once or twice daily. Cows, at least those familiar with housing, will choose to be indoors in certain weather conditions (e.g., periods of greater heat load or rainfall; Legrand et al., 2009).

Although cows may be, for example, cooled with water before afternoon milking (Kendall et al., 2007) or by changing the milking times or frequencies used to decrease heat load (Kendall et al., 2008; Schütz et al., 2023), these solutions may not eliminate the cows' motivation to access shade while at pasture. In addition to the wise utilization of shade through investment in planting and incorporating trees in farm landscapes (e.g., silvopastoral systems; Hendriks et al., 2025), there are potential long-term solutions to mitigate heat stress. This is especially important considering a warming planet and continued breeding for milk production resulting in more heat-sensitive dairy cattle (Kadzere et al., 2002). Solutions include breeding for improved heat tolerance, including for a phenotype with a shorter (slick) coat and reduced hair follicles, as well as an improved ability to adapt to a mild heat load (Littlejohn et al., 2014; Worth et al., 2023).

Although summer may challenge pasture-based animals with periods of excessive heat, so too may cold and wet conditions during winter, when cattle are motivated to seek shelter from wind and rain (Vandenheede et al., 1995; Redbo et al., 2001). Although the features of high-quality shelter are not as well established as for shade, it should protect animals from wind and rain, offer a comfortable (clean, dry, and soft) lying surface (Schütz et al., 2019; Tucker et al., 2021), and be accessible at night, when the majority of lying occurs (e.g., Cartes et al., 2021).

Ensuring cow comfort in winter, especially in large herds managed outdoors, remains a challenge, especially at times when paddocks are wet and muddy, which negatively affects lying times (O'Connor et al., 2019; Neave et al., 2022), cleanliness (Neave et al., 2022; Schütz et al., 2024), and overall welfare (Schütz et al., 2025). Lying on wet surfaces exacerbates the negative effects of wet, windy, and cold weather conditions on thermoregulation. This is especially so among vulnerable and young animals, with rainfall, wet, windy, and cold conditions contributing to increased calf mortality (Diesch et al., 2004; Cuttance et al., 2017). Farmers are currently mitigating the negative effects of winter weather by moving cows to sheltered areas, when possible, by using indoor wintering systems, or by using alternative systems (for example, hay bale grazing; Schütz et al., 2024).

Despite the wealth of information on the impacts of adverse weather on dairy cattle physiology and behavior, as well as varied efforts recommending the provision of shelter to farm animals, it remains an example of what Dwyer et al. (2016) described as “stubbornly unchanging”: the accumulation of knowledge not appearing to improve animal welfare. For example, a survey of pastoral farmers (Fisher et al., 2019) confirmed that the desire to provide livestock with shelter was part of an “innate care for animals, financial considerations and professional pride” (p. 39). However, it was also subject to available resources (e.g., time, money, return on investments), potential impacts on productivity, and having adequate knowledge of initiatives that ameliorated compromises to welfare.

Although cows that spend most of their time at pasture are thought to have greater freedom to express natural behavior, as in any system, they are constrained to some degree. For example, electric fencing, widely used to contain and control the movement of dairy cows at pasture, is beginning to be replaced, at least internally, by virtual fencing (i.e., containing cows to a pasture allocation) and virtual herding (i.e., guiding cows to and from the paddock) on some farms (Fuchs et al., 2025; Verdon et al., 2026). These use punishment to train cattle to respond to a primary audio or vibration cue, with a painful and aversive electric shock delivered if the cue is ignored; both aversive audio cues without shock and reward-based approaches appear ineffective in managing cows at pasture (Wredle et al., 2006; Umstatter et al., 2013; Johansen et al., 2025). An associative learning process requires animals to receive several shocks to learn to respond to the cue and avoid subsequent shocks. The duration of exposure to unpredictable and uncontrollable shocks in training is critical to the animal's welfare. Once trained, the receipt of electric shocks should be both infrequent and under the animal's control.

Quantitative assessments of virtual fencing systems provide some confidence that aversive signals are short-lived and that animals learn quickly. For instance, during training, 61% of cues resulted in shock in the first 7 h, falling to 16% thereafter, with most cows receiving <10 shocks on day one and <1 shock/day after that (Verdon et al., 2024). There is no evidence of increased stress in dairy cows in the days or weeks following training (Hamidi et al., 2022; Fuchs et al., 2024; Verdon et al., 2026). Fast and effective associative learning, then, is key to providing cows with control over the receipt of electric shock, thereby reducing the number of shocks received, restoring predictability and controllability and protecting animal welfare (Lee et al., 2018).

The animal welfare implications of the counterfactual situation for pasture-based systems must also be considered. Although virtual systems always provide a primary cue before delivering electric shock, shocks in the traditional physical electric fencing systems are neither predictable nor controllable when cows cannot see the fence. More than half of shocks are received on or around the head, reflecting the tendency of cows to graze underneath the wire (Verdon et al., 2026). In addition, some virtual systems also use machine learning to identify the lowest shock intensity needed to achieve the desired response for an individual animal. When used appropriately, virtual fencing might, therefore, enhance the animal's grazing experience, reducing inadvertent electric shocks from which the animal receives no warning, but more research is required to understand how the animal experiences these systems.

Along with gains in labor productivity and worker experiences associated with virtual herding, there are anecdotal reports of potential animal health and welfare benefits, including reduced incidence of lameness. Walking long distances to and from milking, farm track maintenance, social dominance interactions, and the degree of patience of the stockperson in herding cows (either on the track or the collecting yard) are possible factors in lameness in pasture-based dairy cows (Chesterton et al., 1989; Sauter-Louis et al., 2004; Ranjbar et al., 2016). As a potential mitigant for lameness, virtual herding may enable less hurried stock management or allow cows to walk in their natural social order or in defined groups that separate dominant cows from submissive herd-mates that are subject to aggressive interactions. By its nature, the technology makes it easier for cows to be moved to shade or shelter in hot or cold wet weather, respectively, while facilitating grazing and allowing for the motivation to seek isolation during calving or facilitating rapid movement to safety in weather-related emergencies, such as flooding. Certain aspects of the technology, including data collation and machine learning algorithms, could enable more precise pasture allocations on a per-cow basis, and perhaps even provide greater opportunities for individual animals to behave as they might prefer to do (e.g., walk in their preferred social order). Conceptually, the system might even offer opportunities for increased cognitive environmental enrichment, as cows have been observed experimenting with the system, for example walking backward or parallel to virtual fence lines to reach extra bites of pasture.

In some ways, virtual systems could represent an advancement over traditional electric fencing, widely accepted in grazing management, but safety mechanisms are needed to prevent uncontrollable or unpredictable receipt of shock. At a minimum, stimuli should not be applied when animals are trotting or running, are stationary for extended periods, at a water trough, or are receiving an unusually high number of shocks. Virtual herding systems should also detect when animals are unable to respond and suspend cues accordingly (e.g., if a laneway is blocked). The potential virtual fencing and herding welfare benefits, challenges, and safeguards associated with this system-level change in cow management deserve further investigation.

In addition to animal welfare, the impact of modern technologies on farm workers, farming, and ultimately society, is a growing area of interest (e.g., Kling-Eveillard et al., 2020; Tuyttens et al., 2022; Kelly et al., 2025). On the one hand, such technology might make work quicker and more effective; on the other hand, it may reduce an individual worker's autonomy or their ability to observe and understand animals. What was once an important contribution to society, animal husbandry, may increasingly be in the hands of technology developers and users. How technologies such as virtual herding are interpreted by various groups remains to be fully explored (e.g., see Grumett and Butterworth, 2022).

Although technologies potentially change the relationship between humans and animals, so too do the actions of those responsible for the husbandry and management of animals (Hemsworth and Coleman, 2011, 2025). Human attitudes and behaviors are not only reflected in animal responses, such as whether they approach or avoid humans, but also influence animal productivity and welfare (Hemsworth et al., 2018; Boivin, 2018; Waiblinger, 2019).

Positive, enriching, and rewarding relationships may buffer the aversive nature of some husbandry procedures, such as the handling and transport of calves (Lensink et al., 2001), whereas negative, fear-provoking, and aversive relationships may result in poor animal health and lowered productivity and reproductive rates (Hixon et al., 1981; Rushen et al., 1999; Breuer et al., 2000). Dependent on the quality, frequency, and context of human–animal interactions (Hinde, 1976), the relationships can be changed by cognitive training that addresses stockperson attitudes and behaviors toward cows (Waiblinger et al., 2006; Hemsworth and Coleman, 2011).

In pasture-based dairying, interactions are concentrated on the dairy, during milking, and in laneways to and from pasture. These are potentially high-risk times for negative interactions, depending on the attitudes and patience of stockpersons. As discussed previously, emerging technologies (e.g., virtual herding) may reduce the risk of negative interactions, though they may also minimize opportunities for observations of animals and positive interactions. Although traits such as ease of handling are valued by stockpersons during unavoidable or necessary interactions (e.g., milking, restraint for health, reproductive treatments), further research is required to understand what interactions, if any, are valued by individual cows. Some animals may seek interactions (e.g., grooming by humans), with others preferring to be left alone; some maybe fearful of novel stimuli, and others interested. The human–animal relationship is commonly framed from a human perspective, yet what cows want, need, or prefer from this relationship, particularly outside of required management interactions such as during grazing, remains largely unknown. Research is required to determine when and where human involvement should be minimized, and when or if positive, cow-centric interactions should be actively promoted.

Driven by Ruth Harrison's devastating 1964 account in *Animal* *Machines* of the lives of calves, pigs, and hens (Harrison, 1964), and the ensuing public debate and governmental report (Brambell, 1965), traditional understandings of animal welfare rightly focus on what animals are experiencing. However, as the pasture-based examples demonstrate, along with what animals are experiencing, understanding their welfare also must consider the actions and perspectives of people. Interpreting what dairy cows feel sometimes requires people to have a more nuanced understanding, such as that of hunger and satiety. Invoking requirements to provide shelter without consideration of the resource requirements and knowledge available to farmers is not likely to see significant change. Technologies have the potential to improve animal welfare and may also provide opportunities and challenges for farmers, and attending to the attitudes and behaviors of animal management staff may not only benefit animal welfare, but also animal production and the human experience.

Such human–animal understandings reflect the multidimensional nature of animal welfare. Animals may suffer as the result of people being unaware, ignorant, or indifferent to animals' needs, or from an inability of those in charge to provide care because they are experiencing their own physical and psychological difficulties. Ill health, difficult financial circumstances or personal and family relationships, adverse climatic conditions (e.g., droughts, blizzards, or floods), and inadequate family or social pastoral support have all been implicated in instances of poor animal welfare (Presland, 2011; Andrade and Anneberg, 2014; Devitt et al., 2015), as have market and economic requirements (Hendrickson and James, 2005; Molnár and Fraser, 2020), including rising costs and falling returns for produce (Fisher, 2018). These are complex biophysical and dynamic social and political issues that sometimes involve conflicting goals.

Because people are at the heart of what happens to farm animals, it is suggested that what is required is to provide the individuals and institutions responsible for the care of animals with the confidence, resources, and opportunities to do what they are, or should be, good at. This may take many forms, such as understanding that animals are at times compromised, for example, by short-term pasture restrictions, which lower milk production and ensure that more pasture of higher quality is available later to maintain higher DMI (Stakelum and Dillon, 1990; Macdonald and Roche, 2024). Or, because people with limited stockperson experience are increasingly joining the livestock production workforce, this highlights the need for nuanced understanding and training, not just of husbandry techniques, but of the impacts of persons' attitudes, beliefs, and behaviors, and their confidence handling animals. Finally, rather than a punitive regulatory approach, exploring the balance between enforcement and encouragement, challenging the culture of farming, and enabling farmers to take ownership of animal welfare, as many do, could result in better outcomes for both animals and humans. For example, regulatory agencies, both governmental and nongovernmental, may need to acknowledge those farms with adequate shelter or with active plans to provide it, as well as those with other justifiable priorities preventing its immediate provision, but also react appropriately to recalcitrant and resistant individuals. Although these suggestions have been largely drawn from consideration of pasture-based dairy farming, they may also apply to other farming systems. Overall, the challenges associated with pasture-based grazing systems are globally relevant, but their magnitude and practical implications are regionally specific and influenced by environmental conditions, animal genetics, and management strategies. Readers are therefore encouraged to interpret the findings of individual studies within their geographic and production contexts.
